# Periprocedural Use of Hypochlorous Acid Mist for Improving Healing and Cosmesis of the Face After Laser

**DOI:** 10.1111/jocd.70412

**Published:** 2025-08-20

**Authors:** Marianna Blyumin‐Karasik, Jessica Colon, Sophie Gaer, Isabella Vigil, Sylvie Nguyen, Jordan Rosen

**Affiliations:** ^1^ Precision Skin & Body Institute Davie Florida USA; ^2^ Nova Southeastern University Dr. Kiran C. Patel College of Osteopathic Medicine Davie Florida USA

**Keywords:** aesthetic, cosmeceuticals, hypochlorous acid, integrated skincare, laser treatment, photoaging, resurfacing laser

## Abstract

**Background:**

Laser resurfacing treatments have revolutionized dermatological procedures by improving skin texture, tone, and quality. Effective periprocedural care remains essential to reduce side effects, support healing, and optimize aesthetic outcomes. Hypochlorous acid (HOCl), a naturally occurring oxidant with anti‐inflammatory and antimicrobial properties, has demonstrated efficacy in promoting wound healing and minimizing scarring.

**Aim:**

To evaluate the use of stabilized HOCl mist in the periprocedural care of patients undergoing laser resurfacing.

**Methods:**

Ten patients underwent treatment with UltraClear, CO2RE, and/or GentleMax Pro laser devices. Stabilized HOCl mist was applied before and after the procedure, with continued application twice daily for 1 week posttreatment. Follow‐up assessments at 1–3 weeks and 1–3 months post‐procedure included clinical photography, tolerability evaluation, and aesthetic outcome assessment. Outcomes measured included Clinician Erythema Assessment (CEA), 4‐point Edema Scale, Investigator Global Assessment of Pigmentation Scale (IPA), and Global Aesthetic Improvement Scale (GAIS). Statistical analysis was performed using the Friedman and Wilcoxon signed‐rank tests.

**Results:**

HOCl mist was associated with accelerated recovery, including a noticeable reduction in erythema and pigmentation. Statistically significant improvements were observed in CEA (*p* = 0.007) and IPA (*p* = 0.012) scores, indicating reduced clinical severity and pigment alteration. Edema and GAIS scores showed no statistically significant change.

**Conclusion:**

Stabilized HOCl mist is well‐tolerated and may significantly aid post‐procedural recovery by minimizing side effects and reducing discomfort. Results support the potential role of stabilized HOCl mist as a beneficial adjunct in post‐laser skincare, contributing to faster healing, reduced inflammation, and enhanced cosmetic results.

## 1 Introduction

Lasers are revolutionizing skin treatments by improving diverse health and aesthetic skin benefits and providing more effective and long‐lasting results [1]. The UltraClear (Acclaro), CO_2_RE (Candela), and GentleMax Pro (Alexandrite, Candela) are highly effective for ablative resurfacing, targeting fine lines, wrinkles, and skin laxity while promoting collagen production, skin tone, textural irregularities and scars, photodamage, and improvement of overall skin quality [2, 3, 4]. The UltraClear is a 2910 nm erbium laser that utilizes ultra‐short pulse technology to deliver precise, controlled resurfacing with minimal thermal damage [2]. CO_2_RE is a 10 600 nm fractional carbon dioxide laser that offers fractional ablative skin resurfacing to stimulate deep tissue remodeling and tightening [3]. The GentleMax Pro utilizes dual wavelengths (755 nm Alexandrite and 1064 nm Nd:YAG) to allow for precise and effective treatments on different skin types and various conditions [4]. In one study, the laser demonstrated efficacy in treating sun damage lentigines, showing improvement in skin photodamage, pigmentation, and overall skin radiance [4].

In order to optimize the results and skin healing after these lasers, peri‐procedure skincare regimens are crucial when utilizing these resurfacing lasers [5]. These include intensive skin moisturization, sun protection, and repair‐promoting cosmeceuticals to reduce downtime and enhance cosmetic outcomes [6]. Proper post‐procedure care accelerates recovery; minimizes side effects; and achieves healthier, brighter, smoother, firmer, and more youthful skin. As the popularity and efficacy of fractional resurfacing laser treatment for skin rejuvenation and acne scarring rise, there is a demand for ideal peri/post‐procedure skin care to enhance healing and cosmesis [7].

Hypochlorous acid (HOCl), an oxidizing molecule produced naturally through the body's innate immune response, has recently gained momentum in dermatology applications for its antimicrobial, anti‐inflammatory, and skin repair properties [8, 9]. Topically applied stabilized HOCl has demonstrated efficacy in creating an optimal wound‐healing environment, potentially reducing scarring after laser procedures [8, 10]. Additionally, HOCl has been effective in treating the compromised skin of atopic dermatitis and associated pruritus, reducing itch and inflammation within 3 days without adverse events [8, 9].

In the context of post‐laser care, HOCl has been incorporated into treatment regimens to enhance healing [11]. A review of post‐laser topical agents highlighted the application of HOCl‐containing sprays multiple times daily during the initial healing phase to support reepithelialization and reduce inflammation post ablative laser resurfacing procedure [12]. Furthermore, studies have shown that certain HOCl solutions increased the degree of reepithelialization by 14% compared with control solutions, indicating its potential to expedite skin wound healing [3, 8, 13]. Additionally, a review panel of leaders in dermatology and plastic surgery has recommended topical HOCl during all phases of injection or laser procedures, from pre‐procedure (removing excess makeup and disinfectant), through peri‐procedure (spraying on the face cold packs) to long‐term post‐procedure (infection prevention and stimulating optimal healing) [14].

Despite these promising findings, there remains a scarcity of evaluations examining the benefits of HOCl application peri‐resurfacing laser procedures. This case series aims to assess the efficacy of peri‐procedure HOCl skin mist in enhancing healing and cosmetic outcomes following treatment with combination and fractional lasers. In this case series, we present 10 patients who underwent a resurfacing laser procedure with peri‐laser application of stabilized HOCl mist.

## 2 Methods and Materials

The study is a case series of 10 patients who underwent fractional UltraClear (Acclaro), CO2RE (Candela), or a combination with Alexandrite (Alex) (GentleMax Pro by Candela) laser procedures on their faces. Table 1 provides information about the patients' demographics, treatment plan, and treatment goal. The laser treatments were performed by a board‐certified dermatologist in a dermatology clinic. Detailed inclusion/exclusion criteria were not implemented as this was designed as a retrospective case series rather than a prospective clinical study. Additionally, informed consent was obtained from all patients.

**TABLE 1:** Patient demographics, treatment plan, and treatment goals.

Patient	Age	Race	Gender	Fitzpatrick skin type	Laser	Treatment	Treatment goal	Used HOCL twice daily
1	59	White	Female	2	UltraClear	*ALEX laser*: 26 J/cm^2^, 3 ms, 2 Hz, 15 mm *UC first treatment*: Clear, hexagon, 15 mm, 30% coverage, 1 ring, 10 μm depth, 0 coag, repeat 0.25 s *UC second treatment*: Clear, hexagon, 15 mm, 30% coverage, 2 rings, 30 μm depth, 0 coag, repeat 0.25 s	Skin rejuvenation	✔
2	48	White	Female	2	UltraClear	*ALEX laser*: 26 J/cm^2^, 3 ms, 2 Hz, 15 mm *UC first treatment*: Laser coring, hexagon, 10 mm, 2% coverage, 0.5 mm depth *UC second treatment*: Laser coring, square, 10 mm, 2% coverage, 1 mm depth *UC third treatment*: Clear, hexagon, 15 mm, 30% coverage, 1 ring, 20 μm depth, 0 coag, repeat 0.25 s	Skin rejuvenation	✔
3	50	White	Female	3	UltraClear	*UC first treatment*: Clear, hexagon, 15 mm, 30% coverage, 1 ring, 30 μm depth, 0 coag, repeat 0.25 s	Skin rejuvenation	✔
4	57	White	Female	2	UltraClear	*ALEX laser*: 28 J/cm^2^, 3 ms, 2 Hz, 12 mm *UC first treatment*: Eye Setting‐Laser coring, hexagon, 7.5 mm, 1% coverage, 0.5 mm depth *UC second treatment*: Around mouth/nose‐ Ultraclear mode, hexagon, 15 mm, 40% coverage, 1 ring, ring depth 40 μm, drill depth 500 μm, 1 coag, 0.25 s *UC third treatment*: Full face‐ clear, hexagon, 15 mm, 30% coverage, 1 ring, 30 μm depth, 0 coag, repeat 0.25 s *UC fourth treatment*: jaw/neck‐ clear, hexagon, 15 mm, 30% coverage, 1 ring, 30 μm depth, no coag, repeat 0.25 s	Skin rejuvenation	✔
5	34	White/Black	Female	4	UltraClear	*UC first treatment*: Clear, hexagon, 15 mm, 30% coverage, 1 ring, 10 μm depth, 0 coag, repeat 0.25 s	Skin rejuvenation	✔
6	38	Asian	Female	3	FCO_2_	FCO_2_ first treatment: 7.6 × 8.9 mm, 5% coverage, 52.9 mJ, repeat 0.5 s	Acne scarring	✔
7	62	White	Female	2	FCO_2_	FCO_2_ first treatment: 7.1 × 8.1 mm, 39% coverage, 3 ring size, 37.1 mJ, repeat 0.25 s	Skin rejuvenation	✔
8	60	White	Female	2	UltraClear	*UC first treatment*: clear, hexagon, 15 mm, 35% coverage, 1 ring, 20 μm, 0 coag, repeat 0.25 s *UC second treatment*: Ultra, hexagon, 15 mm, 2% coverage, 300 μm, 0 coag, repeat 0.25 s	Skin rejuvenation	✔
9	56	White	Female	2	UltraClear	*UC first treatment*: Clear, hexagon, 15 mm, 30% coverage, 1 ring, 30 μm depth, 0 coag, repeat 0.25 s	Skin rejuvenation	✔
10	49	Hispanic	Male	3	UltraClear	*UC first treatment*: Ultra, hexagon, 15 mm, 30% coverage, 300 μm depth, 1 coag, repeat 0.25 s *UC second treatment*: Clear, hexagon, 15 mm, 30% coverage, 1 ring, 10 μm, 0 coag, repeat 0.25 s	Skin rejuvenation	✔

Abbreviations: FCO_2_, fractional CO_2_; UC, ultraclear.

### 2.1 HOCl Mist and Periprocedural Care

Prior to the laser procedure, all participants had topical anesthetic preparation of compounded lidocaine 15% prilocaine/5% phenylephrine 0.5% for 1 h. This was then removed with a 50/50 mix of alcohol and acetone solution.

The HOCl mist used in this case series consists of stabilized 0.02% HOCl, formulated for skin resilience and homeostasis. HOCl mist was applied immediately prior to the laser procedure with gauze. Following the procedure, patients' faces were directly sprayed with HOCl, and they were instructed to spray their faces twice daily with HOCl spray for 1 week following the laser procedure.

The post‐laser regimen also included twice daily application of occlusive biogel (Anteage), as‐needed application of petrolatum‐based emollient, mineral‐based sunscreen, and a gentle facial cleanser nightly for 1 month post‐procedure. Patients were also encouraged to use over‐the‐counter oral antihistamines as needed during their first week post‐laser recovery. They were also guided to moisturize and protect their skin from sun exposure after the healing process for the most sustainable laser results.

### 2.2 Post‐Procedure Healing Follow‐Up Visits

**FIGURE 1:**
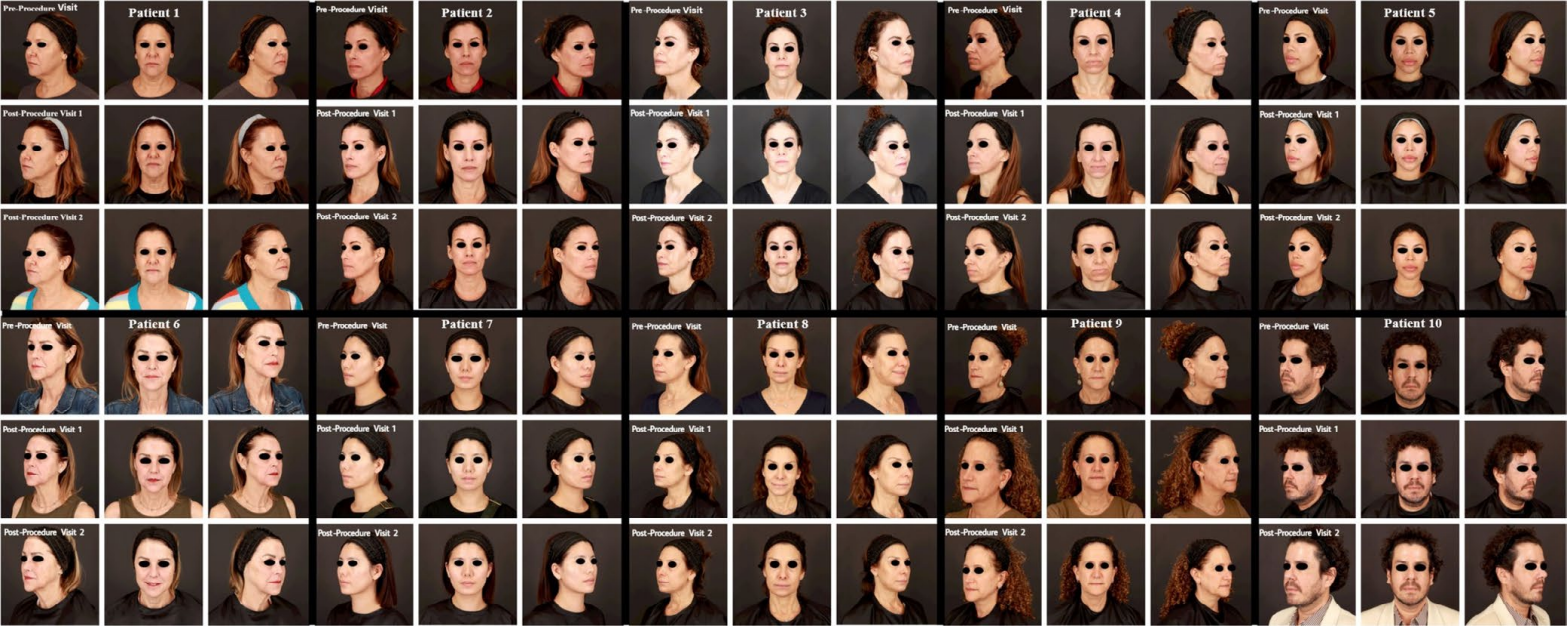
Clinical photographs before and after laser treatment.

Patients received one laser procedure visit followed by two follow‐up visits, each consisting of post‐laser photography (Figure 1) and a brief assessment of the tolerability and cosmetic benefits of the HOCl spray. The first follow‐up visit was 1–3 weeks post‐laser, and the second follow‐up visit was 1–3 months post‐laser.

### 2.3 Pre and Post‐Procedure Skin Assessment

A board certified dermatologist and expert grader, who was blinded to the treatment protocol, specifically the laser procedures performed and the skincare product used, evaluated before and after facial images of each patient using the combined post‐procedure skin assessment: Clinician Erythema Assessment (CEA), 4‐point Edema Scale, Investigator Global Assessment of Pigmentation Scale (IPA), and Global Aesthetic Improvement Scale (GAIS) (Appendix S1).

### 2.4 Data Analysis

Following photo evaluation and completion of the combined post‐procedure skin assessment, the Friedman test was employed to assess differences in CEA, Edema, and IPA scores across Pre‐Treatment, Post‐Treatment Visit 1, and Post‐Treatment Visit 2. To evaluate changes in GAIS score between Post‐Treatment Visit 1 and Post‐Treatment Visit 2, the Wilcoxon signed‐rank test was utilized. No correction for multiple comparisons was applied.

## 3 Results

At the follow‐up visits that occurred at weeks 1–3 post‐laser treatment, patients reported expected skin redness, swelling, focal crusting, and exfoliation of the skin. These symptoms were resolved at the follow‐up visit for all patients. Additionally, they reported that these symptoms lasted for approximately 5 days. All patients reported a good experience with using HOCl Mist, denying skin discomfort before laser treatment and denying worsening of skin redness, swelling, stinging, and burning sensation post‐laser, as well as during the healing process. All patients reported feeling a more “calming or soothing” sensation with the use of HOCl mist as part of their post‐care routine. The results revealed that the post‐laser recovery treatment was well‐tolerated, accelerated recovery, and minimized side effects.

To evaluate changes in patient outcomes across treatment time points, we analyzed four variables: CEA Score, Edema Score, IPA Score, and GAIS Score, at Pre‐Treatment, Post‐Treatment Visit 1, and Post‐Treatment Visit 2 (GAIS Score was assessed only at Post‐Treatment Visits 1 and 2). Descriptive statistics, including median and interquartile range (IQR), were calculated for each variable at each time point. For variables measured at three time points (CEA, Edema, and IPA Scores), the Friedman test was used to assess differences across Pre‐Treatment, Post‐Treatment Visit 1, and Post‐Treatment Visit 2. For the GAIS Score, differences between Post‐Treatment Visit 1 and Post‐Treatment Visit 2 were evaluated using the Wilcoxon signed‐rank test. No correction for multiple comparisons was applied. Statistical significance was defined at *p* < 0.05. Results are summarized in Table 2.

**TABLE 2:** Descriptive statistics and statistical test results for patient scores across treatment time points.

Variable	Time point	Median	IQR	Test	Statistic	*p* value	Significant
CEA score	Pre‐Treatment	2.0	1.5	Friedman	9.879	0.007	Yes
	Post‐Treatment Visit 1	3.0	0.8				
	Post‐Treatment Visit 2	1.0	0.0				
Edema score	Pre‐Treatment	0.0	1.0	Friedman	4.786	0.091	No
	Post Treatment Visit 1	1.0	1.8				
	Post Treatment Visit 2	0.5	1.0				
IPA score	Pre‐Treatment	2.5	1.0	Friedman	8.867	0.012	Yes
	Post Treatment Visit 1	2.0	1.8				
	Post Treatment Visit 2	1.0	0.8				
GAIS score	Post Treatment Visit 1	2.5	2.5	Wilcoxon	0.0	0.062	No
	Post Treatment Visit 2	4.0	1.8				

*Note:* Clinician Erythema Assessment (CEA), 4‐point Edema Scale, Investigator Global Assessment of Pigmentation Scale (IPA), and Global Aesthetic Improvement Scale (GAIS).

CEA Score showed a significant change over time (Friedman statistic = 9.879, *p* = 0.007), with medians of 2.0 (IQR 1.5) at Pre‐Treatment, 3.0 (IQR 0.8) at Post‐Treatment Visit 1, and 1.0 (IQR 0.0) at Post‐Treatment Visit 2. These results reflect the clinical trend: erythema increased post‐treatment for most patients and subsequently resolved by the second follow‐up. Edema Score did not show significant changes (Friedman statistic = 4.786, *p* = 0.091), with medians of 0.0 (IQR 1.0), 1.0 (IQR 1.8), and 0.5 (IQR 1.0) across the respective time points. Three patients demonstrated mild puffiness post‐treatment; however, for the remaining patients, edema either increased post‐treatment and improved by Visit 2 or was absent altogether throughout the post‐treatment period. IPA Score exhibited a significant change (Friedman statistic = 8.867, *p* = 0.012), with medians of 2.5 (IQR 1.0), 2.0 (IQR 1.8), and 1.0 (IQR 0.8), supporting the observed trend of pigmentation reduction in nearly all patients by the final visit.

GAIS Score showed no significant difference between Post‐Treatment Visits 1 and 2 (Wilcoxon statistic = 0.0, *p* = 0.062), with medians of 2.5 (IQR 2.5) and 4.0 (IQR 1.8), respectively. However, individual patient data revealed meaningful clinical improvement: 9 out of 10 patients showed an improved GAIS score by Visit 2, with several patients progressing from “worse” or “no change” at Visit 1 to “much improved” or “very much improved” by Visit 2. Only one patient did not display improvement. Despite these clinical trends, the lack of statistical significance should be interpreted as a limitation of the study. This may reflect not only the small sample size and subjective nature of GAIS but also the potential variability in patient‐reported outcomes that limits the strength of these findings.

## 4 Discussion

Lasers are an important armamentarium in treating a variety of skin health and cosmetic concerns. Ablative and fractional laser resurfacing procedures have emerged as powerful tools in dermatology for improving a range of skin quality parameters, including tone, texture, tightness, and pigmentation irregularities [2, 3, 4]. As these technologies continue to evolve, so too does the importance of optimizing periprocedural care to enhance recovery and aesthetic outcomes. In this context, our case series highlights the potential benefits of incorporating stabilized hypochlorous acid (HOCl) mist into peri‐laser regimens.

HOCl's efficacy lies in its unique biochemical profile. As a naturally occurring oxidant produced by neutrophils, it exerts broad‐spectrum antimicrobial action without inducing cytotoxicity in host tissues [8, 9]. Furthermore, it modulates inflammatory cascades and promotes an optimal environment for wound healing [8, 10]. Importantly, the effectiveness of HOCl is formulation‐dependent, with parameters such as pH and concentration critical to ensuring stability and clinical utility [10, 14]. The formulation used in this study, 0.02% stabilized HOCl with neutral pH, meets the standards outlined in dermatologic literature for optimal efficacy and tolerability. This may account for the high patient satisfaction and absence of irritation or delayed healing observed in our case series.

The results from our cohort of 10 patients suggest that the use of HOCl mist is not only well‐tolerated but may also play a meaningful role in expediting post‐procedural healing, reducing subjective discomfort, and minimizing common adverse effects such as erythema and edema. All patients reported subjective improvement in comfort during the immediate post‐laser period, stating sensations of “soothing” and “calming” upon application of the mist. These findings are consistent with previously published literature that identifies HOCl's anti‐inflammatory, antimicrobial, and wound healing capabilities as advantageous in peri‐laser settings [8, 9, 10].

Quantitative analysis supports these observations as significant improvements were seen in both CEA and IPA scores over time, reflecting enhanced clinical outcomes and improved patient‐reported assessments of irritations and procedural aftereffects. Notably, CEA scores demonstrated a clinical pattern in which erythema increased after treatment for most patients before resolving by the second follow‐up, suggesting a typical inflammatory response that was effectively controlled. While edema scores showed a nonsignificant trend toward improvement, this may reflect variability in individual inflammatory response. Three patients had mild puffiness post‐treatment, whereas others either experienced edema that resolved by the second visit or had no measurable edema throughout the post‐treatment period. Additionally, nearly all patients experienced an improvement in pigmentation over time, consistent with the significant changes in IPA scores. This supports the observed trend of pigmentation reduction in nearly all patients by the final visit. Although GAIS scores improved numerically from Visit 1 to Visit 2, the change did not reach statistical significance, potentially due to the small sample size or the subjective nature of aesthetic assessments. However, 9 out of 10 patients demonstrated improvement by the final follow‐up, with several showing marked or very much improved scores, further reinforcing the favorable clinical trajectory observed. These findings highlight a positive clinical trend, even in the absence of statistical significance, indicating that patients had visible improvements in skin appearance and perceived cosmetic outcomes over time. Overall, the combination of positive patient‐reported outcomes and statistically significant improvements in key clinical parameters highlights HOCl mist as a promising adjunct in peri‐laser skincare protocols.

Our observations also support the broader trend in procedural dermatology advocating for holistic peri‐care regimens. Pre‐procedure preparation (including skin cleansing and antisepsis) and post‐procedure maintenance (emollients, gentle cleansers, and photoprotection) are essential components in reducing complication rates such as infection and post‐inflammatory hyperpigmentation, as well as contributing to skin repair and appearance.

HOCl, with its multifunctional profile, integrates seamlessly into each phase of the skin recovery continuum, from pre‐treatment cleansing and antisepsis to post‐treatment inflammation, infection prevention, and wound healing support. According to Panossian, adaptogens are defined as innocuous agents that increase the state of nonspecific resistance to stress, enhance the body's ability to adapt to external stressors, and normalize via multiple physiological functions [15]. Based on this definition, and supported by Liu et al., who identified and characterized agents with adaptogenic properties in dermatologic contexts, HOCl may be categorized as an active skin adaptogen [16]. Its nonhazardous nature, multimodal efficacy, and role in maintaining skin homeostasis further reinforce this classification. Evidence has demonstrated that skin adaptogens offer several normalizing advantages, such as enhancing resistance to stress, diminishing inflammation, improving skin repair physiology, and delaying premature aging [17]. Thus, as a skin adaptogen, HOCl possesses the ability to assist the skin's return to a healthier state of equilibrium after a laser procedure.

Although the small sample size, short‐term follow‐up, and lack of a control group limit the generalizability of our findings, the uniform positive patient experiences and favorable healing observations provide a compelling foundation for further study. Additionally, a randomized, controlled trial comparing HOCl mist to placebo or alternative post‐laser topical agents would be invaluable in identifying its true clinical advantage.

This case series supports the adjunctive use of stabilized HOCl mist in the periprocedural management of laser procedures. With a favorable safety profile, ease of use, and multifaceted therapeutic action, HOCl mist represents a promising addition to the peri‐laser skin care toolkit. Our initial results suggest that HOCl may contribute meaningfully to enhanced healing, reduced complications, and improved patient satisfaction following laser treatments.

## Author Contributions

M.B.‐K. and J.C. contributed to the drafting and editing of the manuscript. S.G. and I.V. contributed to the participants' data collection and entry. S.N. contributed to the data analysis, and J.R. performed the blinded photo evaluation. All authors have read and approved the final manuscript.

## Ethics Statement

We would like to extend gratitude to the patients participating in the study, who were treated in the most ethical way in accordance with Good Clinical Practices.

## Consent

All the patients participating in the study agreed and signed the photo release consent for their photography for this publication.

## Conflicts of Interest

Marianna Blyumin‐Karasik, MD is a founder and CEO of Stamina Beauty LLC, manufacturer of HOCl mist.

## Supporting information


**Appendix S1:** jocd70412‐sup‐0001‐AppendixS1.pdf.

## Data Availability

The data that support the findings of this study are available from the corresponding author upon reasonable request.
