# Supplementary material for: Periprocedural Use of Hypochlorous Acid Mist for Improving Healing and Cosmesis of the Face After Laser
Source: J Cosmet Dermatol. 2025 Aug 20;24(8):e70412. doi: 10.1111/jocd.70412 (PMC12365733; doi:10.1111/jocd.70412)
Supplement: Supplementary file 1 — Appendix S1: jocd70412‐sup‐0001‐AppendixS1.pdf. [file JOCD-24-e70412-s001.pdf]

Patient Number: ____________

Pre-Treatment Photonumeric Score

Clinician Erythema Assessment (CEA): select the appropriate description of the patient's facial redness.

Clinicians Erythema Assessment	Score
Clear Skin with no signs of erythema	0
Almost clear; slight redness	1
Mild erythema, definite redness	2
Moderate erythema; marked redness	3
Severe erythema; fiery redness	4

Clinician Erythema Assessment Score: _______

4-point Edema Scale: select the appropriate description of the patient's facial edema.

4-point Edema Scale	Score
Clear, no edema	0
Mild Puffiness	1
Moderate Puffiness	2
Marked Severe Edema	3

4-point Edema Scale Score: _______

IGA Pigmentation Scale: select the appropriate description of the patient's pigmentation severity.

Score	Descriptors
0	Clear of dyspigmentation
1	Almost clear of dyspigmentation
2	Mild, but noticeable dyspigmentation
3	Moderate dyspigmentation (medium brown in quality)
4	Severe dyspigmentation (dark brown in quality)
5	Very severe dyspigmentation (very dark brown, almost black in quality)

IGA Pigmentation Score: __________

Investigators Initials: _________ Date: ___________

Patient Number: ____________

Post-Treatment Photonumeric Score Visit 1

Clinician Erythema Assessment (CEA): select the appropriate description of the patient's facial redness.

Clinicians Erythema Assessment	Score
Clear Skin with no signs of erythema	0
Almost clear; slight redness	1
Mild erythema, definite redness	2
Moderate erythema; marked redness	3
Severe erythema; fiery redness	4

Clinician Erythema Assessment Score: _______

4-point Edema Scale: select the appropriate description of the patient's facial edema.

4-point Edema Scale	Score
Clear, no edema	0
Mild Puffiness	1
Moderate Puffiness	2
Marked Severe Edema	3

4-point Edema Scale Score: _______

IGA Pigmentation Scale: select the appropriate description of the patient's pigmentation severity.

Score	Descriptors
0	Clear of dyspigmentation
1	Almost clear of dyspigmentation
2	Mild, but noticeable dyspigmentation
3	Moderate dyspigmentation (medium brown in quality)
4	Severe dyspigmentation (dark brown in quality)
5	Very severe dyspigmentation (very dark brown, almost black in quality)

IGA Pigmentation Score: _________

Investigators Initials: _________ Date: ____________

Patient Number: _____________

Global Aesthetic Improvement Scale (GAIS): select the appropriate description of the patient's global aesthetic improvement.

**Rating**		**Description**
1	Worse	The appearance is worse than the original condition.
2	No change	The appearance is essentially the same as the original condition.
3	Improved	Obvious improvement in appearance from the initial condition, but a re-treatment is indicated.
4	Much improved	Marked improvement in appearance from the initial condition, but not completely optimal for this subject.
5	Very much improved	Optimal cosmetic results in this subject

Global Aesthetic Improvement Scale Score: ____________

Investigators Initials: __________ Date: ____________

Patient Number: ____________

Post-Treatment Photonumeric Score Visit 2

Clinician Erythema Assessment (CEA): select the appropriate description of the patient's facial redness.

Clinicians Erythema Assessment	Score
Clear Skin with no signs of erythema	0
Almost clear; slight redness	1
Mild erythema, definite redness	2
Moderate erythema; marked redness	3
Severe erythema; fiery redness	4

Clinician Erythema Assessment Score: _______

4-point Edema Scale: select the appropriate description of the patient's facial edema.

4-point Edema Scale	Score
Clear, no edema	0
Mild Puffiness	1
Moderate Puffiness	2
Marked Severe Edema	3

4-point Edema Scale Score: _______

IGA Pigmentation Scale: select the appropriate description of the patient's pigmentation severity.

Score	Descriptors
0	Clear of dyspigmentation
1	Almost clear of dyspigmentation
2	Mild, but noticeable dyspigmentation
3	Moderate dyspigmentation (medium brown in quality)
4	Severe dyspigmentation (dark brown in quality)
5	Very severe dyspigmentation (very dark brown, almost black in quality)

IGA Pigmentation Score: _________

Investigators Initials: _________ Date: ___________

Patient Number: ____________

Global Aesthetic Improvement Scale (GAIS): select the appropriate description of the patient's global aesthetic improvement.

**Rating**		**Description**
1	Worse	The appearance is worse than the original condition.
2	No change	The appearance is essentially the same as the original condition.
3	Improved	Obvious improvement in appearance from the initial condition, but a re-treatment is indicated.
4	Much improved	Marked improvement in appearance from the initial condition, but not completely optimal for this subject.
5	Very much improved	Optimal cosmetic results in this subject

Global Aesthetic Improvement Scale Score: ___________

Investigators Initials: _________ Date: ___________
